# Method for Rapid Analysis of Mutant RNA Polymerase Activity on Templates Containing Unnatural Nucleotides

**DOI:** 10.3390/ijms22105186

**Published:** 2021-05-14

**Authors:** Tatiana Egorova, Ekaterina Shuvalova, Sabina Mukba, Alexey Shuvalov, Peter Kolosov, Elena Alkalaeva

**Affiliations:** 1 Engelhardt Institute of Molecular Biology, The Russian Academy of Sciences, 119991 Moscow, Russia; tatvladegorova@gmail.com (T.E.); hritova_katia@mail.ru (E.S.); sabina.mukba1996@gmail.com (S.M.); laursen1243@mail.ru (A.S.); 2 Institute of Higher Nervous Activity and Neurophysiology, The Russian Academy of Sciences, 117485 Moscow, Russia

**Keywords:** unnatural nucleotides, T7 RNAP, cell-free translation

## Abstract

Pairs of unnatural nucleotides are used to expand the genetic code and create artificial DNA or RNA templates. In general, an approach is used to engineer orthogonal systems capable of reading codons comprising artificial nucleotides; however, DNA and RNA polymerases capable of recognizing unnatural nucleotides are required for amplification and transcription of templates. Under favorable conditions, in the presence of modified nucleotide triphosphates, DNA polymerases are able to synthesize unnatural DNA with high efficiency; however, the currently available RNA polymerases reveal high specificity to the natural nucleotides and may not easily recognize the unnatural nucleotides. Due to the absence of simple and rapid methods for testing the activity of mutant RNA polymerases, the development of RNA polymerase recognizing unnatural nucleotides is limited. To fill this gap, we developed a method for rapid analysis of mutant RNA polymerase activity on templates containing unnatural nucleotides. Herein, we optimized a coupled cell-free translation system and tested the ability of three unnatural nucleotides to be transcribed by different T7 RNA polymerase mutants, by demonstrating high sensitivity and simplicity of the developed method. This approach can be applied to various unnatural nucleotides and can be simultaneously scaled up to determine the activity of numerous polymerases on different templates. Due to the simplicity and small amounts of material required, the developed cell-free system provides a highly scalable and versatile tool to study RNA polymerase activity.

## 1. Introduction

The genetic code can be modified at different levels, namely nucleotides, codons, and amino acids. At each of these levels, unnatural components can be used. Thus, unnatural amino acids can be encoded by unnatural nucleotides within the three-letter codons [1,2,3,4,5], four-letter codons [6], or by reassignment of sense or stop codons [7,8,9].

The development of unnatural base pairs (UBPs) is one of the most remarkable advances in synthetic biology. UBPs, which function as a third base pair in replication, transcription, and translation, expand the genetic alphabet and can be used in various applications such as site-specific fluorescence labeling, nucleic acid immobilization, and DNA and RNA analysis. Originally synthesized to expand the genetic alphabet, UBPs have become powerful tools for monitoring the structural changes in nucleic acids and their interactions with other molecules [10,11,12,13,14,15,16,17,18,19,20,21,22].

All UBPs obtained to date can be divided into two groups: (1) UBPs, which form hydrogen bonds between bases (hydrogen complementarity) and (2) UBPs with hydrophobic interactions within the pair (structural complementarity) [23,24,25,26,27,28,29,30,31,32,33,34,35,36]. The first synthesized UBPs comprised hydrogen bonds. Their topology differed from that found in natural nucleotides, which provided specificity for enzymatic synthesis [23,24,25,26,27,28,29,30,31]. Later, hydrophobic UBPs were obtained [32,33,34,35,36]. This proved that hydrogen bonding is not the only mechanism capable of storing and transmitting genetic information. Presently, both types of UBPs are being actively developed and improved; however, for the amplification and transcription of templates containing unnatural nucleotides, the presence of DNA and RNA polymerases capable of their recognition is required. Under favorable conditions, in presence of modified nucleotide triphosphates, DNA polymerases synthesize unnatural DNA with high efficiency [17,37,38,39,40,41]; then with RNA polymerases, everything is not so good. At present, the available RNA polymerases for in vitro studies are specific to natural nucleotides and do not recognize unnatural nucleotides [31,42,43,44,45].

Bacteriophage T7 RNA polymerase (T7 RNAP) is one of the widely used polymerases for incorporating UBPs into RNA. It is a one subunit protein with 883 amino acid residues (98 kDa). T7 RNAP is capable of performing a full transcription cycle without involving any additional protein factors [46]. For a better understanding of the mechanisms underlying transcription, experiments on T7 RNA polymerase mutagenesis were carried out. As a result of this mutational analysis, it was found, for example, that the Y639F mutant has an amazing ability to use both rNTP and dNTP as substrates [47]. The additional mutation S641A markedly enhanced this property; therefore, the “double” mutant Y639F/S641A was able to synthesize long mixed polynucleotides, where one, two, or three types of rNTPs were replaced by the corresponding dNTPs [48,49]. Moreover, mutant RNA polymerase is used in the development of synthetic nucleotides. For example, some mutant variants of T7 RNAP, such as Y639F, Y639F/H784A, and G542V/H772R/H784S, were obtained to increase the efficiency of incorporating pyrimidine-2′-modified nucleoside triphosphates used for site-specific labeling of RNA [50].

Nevertheless, presently, no simple methods are available for determining the activity of mutant forms of RNA polymerase, synthesizing RNA on a template containing UBPs. In the present study, we proposed a new method based on a double-coupled (coupled^2^) cell-free transcription–translation system, in which we determined the activity of mutant T7 RNA polymerases by the luminescence of synthesized nanoluciferase (Nluc). As a result, we evaluated the transcription efficiency of unnatural templates with different mutants of T7 RNA polymerases. In our system mutant T7 RNAPs used natural ribonucleotides to transcribe DNA templates containing UBPs. Furthermore, we revealed high sensitivity and simplicity of the developed method and demonstrated the ability of the studied mutants of T7 RNA polymerase to recognize UBPs. Additionally, we demonstrated that the approach we developed for determining the activity of RNA polymerases is applicable to a wide range of UBPs.

## 2. Results

### 2.1. Overview of the Method

**Figure 1:**
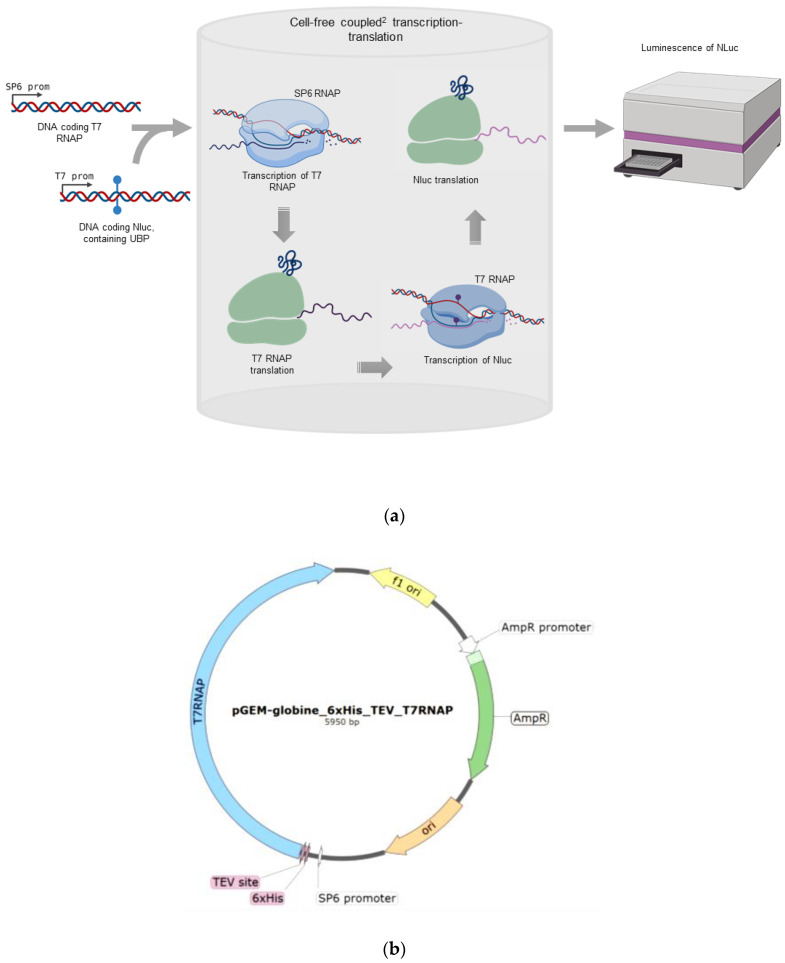
Cell-free “coupled^2^” transcription–translation system. (**a**) Scheme of the cell-free “coupled^2^” transcription–translation system. (**b**) Map of the plasmid encoding the human beta-globin 5′UTR, the T7 RNAP coding sequence containing a 6xHis tag, and a subsequent TEV protease site at the N-terminus.

For simple and efficient testing of the functional activity of mutant T7 RNA polymerases, we developed a method of a coupled^2^ transcription–translation system. The scheme of the method is illustrated in Figure 1. SP6 RNA polymerase together with a linear template for T7 RNA polymerase containing the SP6 promoter and linear template for Nluc containing the T7 promoter was added to a wheat germ extract (WGE) cell-free system for coupled transcription and translation. NanoGlo, a substrate for Nluc, was also added to the reaction. First, SP6 RNA polymerase from the SP6 promoter synthesized mRNA for translation of T7 RNA polymerase, thereby producing T7 RNAP. The newly formed T7 RNAP enzyme transcribed Nluc mRNA from the T7 promoter, and thereafter, Nluc was translated in the same lysate leading to the formation of a luminescent product. This allowed us to determine the activity of T7 RNA polymerase in one test tube, starting from the DNA encoding it, without using difficult methods to obtain and purify RNA and proteins. The addition of the Nluc DNA template containing UBPs allowed us to study the ability of T7 RNAP to use an unnatural template to synthesize mRNA with natural ribonucleotide triphosphates.

We selected Nluc as a reporter protein for determining the functional activity of mutant T7 RNA polymerases for the following reasons: (1) luciferases induce luminescence in the presence of substrate immediately after their translation because they do not need a maturation time and they leave the ribosome in a catalytically active form [51]; (2) among all known luciferases, we selected Nluc, since it not only has three times smaller size than the widely used firefly and Renilla luciferases, but also causes 100 times higher luminescence levels than classical luciferases [52]. Thus, the RNA of this protein is translated faster than that of other luciferases, while the method for determining the activity of mutant T7 polymerases is more sensitive by two orders of magnitude, which is crucial for our task.

### 2.2. Testing of T7 RNAP Mutants on a Natural Template

To investigate the transcriptional efficiency of T7 RNAP, we constructed a plasmid encoding the human beta-globin 5′UTR, T7 RNAP coding sequence containing a 6xHis tag, and a subsequent TEV protease site at the N-terminus (Figure 1b). The globin leader provides an efficient translation of mRNA even in the absence of a 5′-cap. The 6xHis tag allows the isolation of T7 RNA polymerase from the lysate. The mRNA obtained from this construct was tested in various lysates and revealed high activity (data not shown).

Along with wild-type T7 RNA polymerase (T7wt RNAP), we obtained by site-directed mutagenesis and tested the functional activity of 10 previously published mutant T7 RNAPs with different specificities (Table 1). As a negative control in our experiments, we used the mutant T7 RNAP containing Leu229UAG replacement (ΔT7 RNAP). Such polypeptide can be synthesized in a cell-free system but cannot transcribe RNA.

For the initial testing of the approach, we first used a Nluc coding template containing only natural dNTPs. To determine the optimal conditions for the coupled^2^ system, we added 1–10 ng/µL of linear DNA coding T7 RNAP to TNT WGE along with ribonucleotide triphosphates (NTPs), SP6 RNA polymerase, and 1 ng/µL DNA encoding Nluc. We observed the maximum luminescence signal using 10 ng/µL linear DNA coding T7 RNAP. The optimized concentrations of the coupled^2^ system are summarized in Table 2.

**Figure 2:**
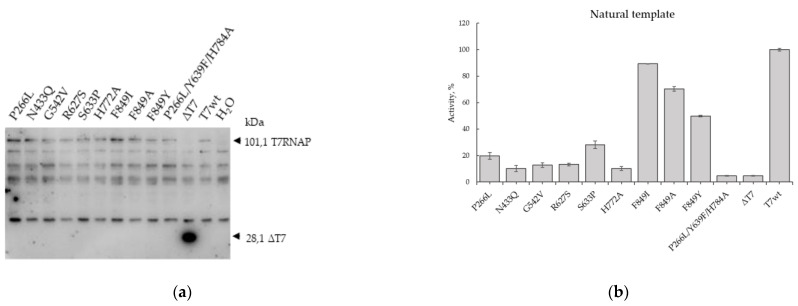
Activity of T7 RNAP mutants on the natural template. (**a**) Expression level of T7 RNAP mutants in the coupled^2^ cell-free system. Western blot of the WGE lysate where T7 RNAP mutants were synthesized. (**b**) Activity of the mutants of T7 RNAP normalized on the amount of T7 RNAP synthesized in the coupled^2^ cell-free system using natural template, *n* = 3, mean ± SD.

After optimizing the experimental conditions, we characterized 10 mutant T7 RNAPs (Table 1) by their ability to transcribe Nluc RNA on a natural DNA template. Following our protocol (Table 2), we incubated linear DNA templates for mutant T7 polymerases in a coupled^2^ system. The T7 RNAP translation was controlled by western blotting with anti-His-tag antibodies (Figure 2a). We have shown that in all samples where PCR products encoding T7wt RNAP and T7 RNAP mutants were added, 101.1 kDa proteins were detected, corresponding to the estimated size of T7 RNAP with tags. In the sample containing the DNA of ΔT7 RNAP, comprising the first 228 codons, the corresponding 28.1 kDa protein product was detected. In the assay where mQ was added instead of T7 RNAP DNA, only proteins nonspecifically interacting with anti-His-tag antibodies were detected. Thus, we have proved that T7wt RNAP and its mutants are specifically synthesized in a cell-free system.

The activities of the mutants during transcription of Nluc mRNA are illustrated in Figure 2b. The amount of synthesized Nluc reflects the amount of transcribed mRNA encoding Nluc, which in turn reflects the activity of the T7 RNAP mutant. All activities of T7 RNAP mutants were normalized to the amounts of the synthesized proteins in the coupled^2^ system determined by western blotting of lysates. We revealed that in this system, one mutant form P266L/Y639F/H784A completely lost its activity on natural mRNA, and the luminescence level in the presence of this mutant was similar to the luminescence induced by the truncated form ΔT7 RNAP. Previously, it was reported that this mutant has increased thermal stability and can accept 2′-O-methyl triphosphates and dNTPs [54]. Moreover, this mutant of T7 RNAP was used to transcribe DNA containing eight nucleotide letters, four natural letters (dG, dC, dA, and dT), and four additional letters (dP, dZ, dS, and dB) [31]. Thus, we revealed that the increased specificity for dNTPs and unnatural nucleotides of this mutant markedly reduced its ability to transcribe natural templates. Presumably, Nluc mRNA synthesized by this mutant contains deoxynucleotides and cannot be utilized by the ribosome during translation in a cell-free system; hence, no luminescence was detected. Most of the other tested mutant forms (P266L, N433Q, G542V, R627S, S633P, H772A) decreased their activity in the coupled^2^ system to 10%–30%. For all these mutants, increased thermal stability was demonstrated earlier [50,54,55,56]. Furthermore, for G542V the violation of interaction with 2′-OH of ribonucleotides was demonstrated [50] and for R627S, the violation of specificity expressed in the incorporation of ddNTP to RNA was shown [54,55]. Additionally, for the mutants S633P and H772A, relative activities in vitro by fluorescent beacon were determined, which were 56% and 70%, respectively [56]. In the coupled^2^ system, we observed the lower activities of these mutants by 30% and 10%, respectively; however, mutants with replacements in position F849 retained more than half of the activity of the T7wt RNAP in the coupled^2^ system—F849I, F849A, and F849Y. For F849I and F849Y mutants, the relative in vitro activities were also determined by fluorescent beacon as 88% and 37%, respectively [56], which is in accordance with our data of 90% for F849I and 50% for F849Y (Figure 2b). Thus, characterizing the activity of T7 RNAP mutants, we confirmed the efficiency of the developed method on the natural DNA template apart from certain discrepancies with the published data, which are presumably bound to the greater proximity of the coupled^2^ system to the natural conditions.

### 2.3. Obtaining DNA Templates Containing UBPs and Testing of T7 RNAP Mutants Activities

The linear DNA template for testing mutant forms of T7 RNA polymerase comprised a promoter for SP6 RNA polymerase, human beta-globin 5′UTR, a sequence encoding an Nluc protein of approximately 600 nucleotides in size, and a polyT tract for obtaining a polyA tail in mRNA. The total size of the PCR product was approximately 800 nucleotides. While choosing a codon for introducing UBP, we focused on the data for critical amino acids in Nluc [58]. Therefore, we selected the second position in the Val29 codon. Additionally, we chose the second position of the valine codon as it can be recognized by four different tRNAs with any nucleotide in the third position. So insertion of inappropriate nucleotide in the second position will change Val codon to another one (Ala, Asp, Glu, or Gly) what will lead to disruption of Nluc activity that can be detected as luminescence decrease. Two types of UBPs were tested in our study: the hydrophobic pair 5-Me-isodC–isodG and the hydrophilic pair dNaM–d5SICS.

**Figure 3:**
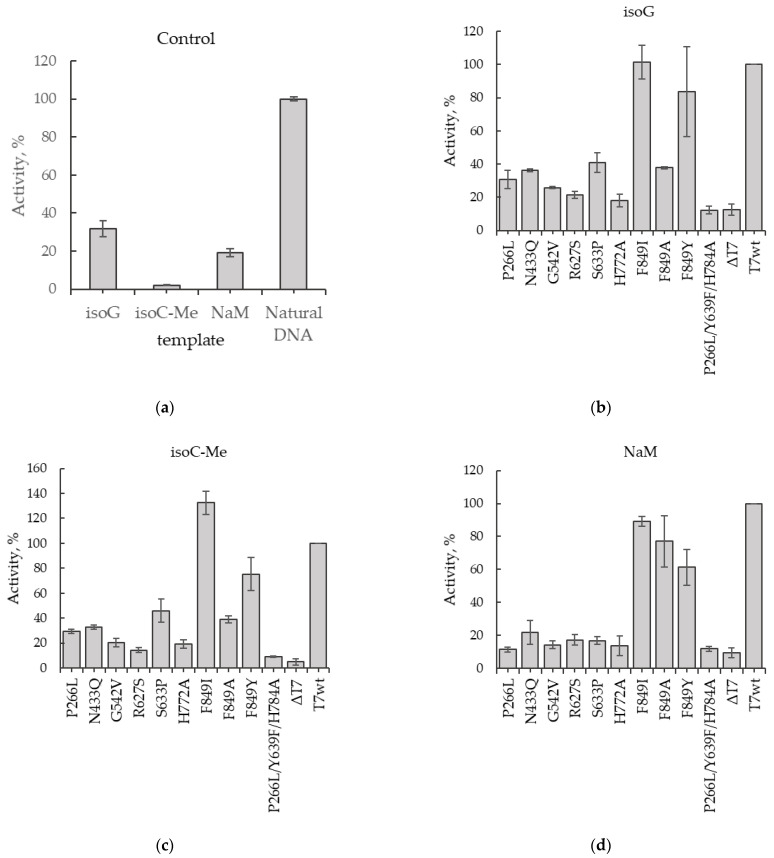
Activity of the T7 RNAP mutants on the templates containing unnatural nucleotides in the second position of V29 codon of Nluc normalized on the amount of T7 RNAP synthesized in the coupled^2^ cell-free system, *n* = 3, mean ± SD. (**a**) Activity of T7 RNAP wt on different templates. (**b**) Activity of T7 RNAP mutants on the template containing isodG. (**c**) Activity of T7 RNAP mutants on the template containing 5-Me-isodC. (**d**) Activity of T7 RNAP mutants on the template containing dNaM.

Initially, we tested the activity of T7wt RNAP at each of the three obtained templates carrying unnatural nucleotides and compared it with the activity of T7wt RNAP at the natural template (Figure 3a). It is important to note that in the transcription reaction with T7 RNAP we used only natural ribonucleotide triphosphates. So RNAP recognized UBP in the DNA template and inserted into RNA natural ribonucleotide. Thus, RNA polymerase was mistaken in reading the unnatural nucleotide in DNA and used available natural ribonucleotides to build RNA. Therefore, the efficiency of transcription with unnatural templates was significantly lower than with natural ones. We observed that on the template containing isodG, the activity of T7wt RNAP was 30%, compared to that of the natural template. Moreover, T7wt RNAP is practically unable to transcribe the template containing 5-Me-isodC. The T7wt RNAP activity on this template was less than 5% compared to the natural one. T7 RNAP activity on the template containing dNaM was approximately 20% of its activity on the natural template.

Further, the activity of the selected mutant RNAPs (Table 1) was determined using unnatural templates. The activity of the mutants was normalized to the activity of T7wt RNAP on the same unnatural templates. On the template with isodG in the second position of the Val29 Nluc codon, the activities of most of the tested mutant RNAPs were lower than that of T7wt RNAP (Figure 3b); however, those of the mutants F849I and F849Y were comparable to the activity of T7wt RNAP on this template. A similar pattern was observed for the unnatural template comprising 5-Me-isodC in the same position. Most of the mutants demonstrated low activity, and only F849I and F849Y mutations had relatively high transcriptional activity (Figure 3c). On the template containing dNaM, the activity of mutants F849I, F849A, and F849Y was comparable to that of T7wt RNAP, and the mutants R627S, N433Q, S633P, G542V, and H772A were less active (Figure 3d). The activity of the mutant P266L/Y639F/H784A was similar to the level of the negative control on all templates; thus, this mutant was unable to transcribe functional mRNA. The high activities of the mutants F849I and F849Y compared to T7wt RNAP on all tested unnatural nucleotides indicated a lower selectivity of these polymerases, which can be used for further designing the mutant RNAPs for obtaining the protein utilizing unnatural nucleotides with high specificity.

Thus, we have demonstrated the efficiency of the developed method and compared the activities of the ten mutant T7 RNAPs on different unnatural templates.

## 3. Discussion

We describe the “coupled^2^” cell-free transcription–translation system, a simple in vitro method to determine RNAP activity on templates containing unnatural nucleotides using a luminescence microplate reader. This method has a major advantage over other approaches, as it does not require expression and purification of proteins to test their activity during transcription. Moreover, in this system, all manipulations are performed in one tube/well for only 1 h. This method allows rapid measurement of activities of different ferments simultaneously in one 364 well microplate. Importantly, the “coupled^2^” system can provide comparable results with other methods such as classical in vitro transcription, thereby suggesting that it is a robust and convenient framework for developing more complex methods.

“Coupled^2^” system has certain advantages over the classical approach, in which a purified recombinant polymerase is used for in vitro transcription. Indeed, classical approaches might not be convenient due to the additional difficult and time-consuming steps of bacterial expression and purification of RNA polymerases. This issue was resolved by using a coupled^2^ cell-free transcription–translation system. As a result, the first round of transcription and translation RNA polymerases can immediately transcribe mRNA in the same tube. A key analytical advantage of this method is that it enables in determining the relative activity of RNAPs based on the luminescence induced by synthesized Nluc, which represents the amount of active RNAP in solution. Thus, this approach allowed easy and reproducible quantitative evaluation of RNAP activities. Furthermore, the use of a microplate reader remarkably simplified the method, which in turn allowed massive parallel analysis of dozens of samples in one day.

Another significant advantage of this method is the possibility to use templates containing different unnatural nucleotides or the use of different transcription activators/suppressors in parallel experiments. This approach might be useful in comparing the RNAP preferences of different unnatural nucleotides and the effects of other transcription regulators. The high sensitivity of the method allows comparison of a high range of RNAP activities, which is particularly important in experiments with components that significantly reduce the transcription activity.

Nevertheless, the coupled^2^ system has certain limitations. Firstly, this is an indirect method; the determination of the activity of RNA polymerases occurs through the translation of the Nluc. This means that in order to obtain unambiguous results, it is necessary to control three parameters: translation of RNA polymerase, Nluc mRNA synthesis, and Nluc mRNA quality. Translation of RNA polymerase could be controlled by the Western blot hybridization, as we did in our work (Figure 2a). The synthesis and the amount of Nluc mRNA can be controlled using either Northern blot hybridization, or by an affinity chromatography purification of newly synthesized RNA polymerase and following in vitro transcription of mRNA. This approach also can be used to control the quality of transcribed mRNA. The quality of mRNA can be analyzed using cDNA sequencing. Therefore, described limitations are not critical and after the appropriate solution of them, they will provide additional information about the activity of the RNAP mutants.

In the future, this approach can be used to quickly determine the activity of RNA polymerases on new substrates and templates, which is presently lacking the development of useful unnatural base pairs within the synthetic approach.

## 4. Materials and Methods

### 4.1. Obtaining Constructs Encoding Mutant Forms of T7 RNAP

T7 RNAP cds was amplified by PCR with Q5 polymerase (NEB, Ipswich, MA, USA) from the plasmid pAR1219 using primers T7_HindIII_F and T7_XhoI_R (Table 3). The resulting PCR product of T7 RNAP and globine 5′UTR were inserted into the pGEM vector (Promega, Madison, WI, USA) at the HindIII and XhoI restriction sites. So we got the basic construction pGEM-globine_6xHis_TEV_T7RNAP. Based on this construct, mutants with single amino acid substitutions and a mutant with a premature stop codon were obtained using the QuikChange Site-Directed Mutagenesis Kit (Agilent, Santa Clara, CA, USA), as well as the P266L_Y639F_H784A mutant using the QuikChange Multi Site-Directed Mutagenesis Kit (Agilent, Santa Clara, CA, USA).

### 4.2. T7 RNAP and Nluc PCR Products Obtaining

The linear templates for T7 RNAP and Nluc were obtained by PCR with the primers shown in Table 3. Reverse primers contained poly(T) to generate a polyadenylated 3′end of mRNAs. The reaction mixture contained 10 ng of plasmids (10 of T7 RNAP mutants, T7_wt, ΔT7 or globine Nluc), 1.25U of GoTaq DNA polymerase (Promega, Madison, WI, USA), Taq buffer, 0.2 mM of each deoxyribonucleotide (dATP, dGTP, dCTP, dTTP) (Thermo Fisher Scientific, Waltham, MA USA), 1 µM of forward and reverse primers in a total volume of 50 µL.

### 4.3. The Coupled^2^ Transcription-Translation System

TnT^®^ T7 Coupled Wheat Germ Extract System (Promega, Madison, WI, USA) was used to perform the transcription and translation of wt and mutant T7 RNAP and then to detect their activity in the Nluc DNA transcribing. Ten T7 RNAP mutants (Table 1), T7wt RNAP, ΔT7 RNAP were tested at the Nluc DNA template, containing natural nucleotides, and three Nluc DNA templates containing the unnatural nucleotides in the second position of V29 codon: isodG, 5-Me-isodC and dNaM. The coupled transcription–translation mixture containing the components described in Table 2 was assembled on ice. The Nluc luminescence signal was detected using a microplate Tecan F200 PRO reader (Tecan Trading AG, Menedov, Switzerland) at 30 °C for 1 h.

### 4.4. Western-Blot Analysis

Amounts of synthetized His-tagged T7 RNAPs or ΔT7 were determined after coupled transcription-translation reaction with DNA fragments encoded T7 RNAPs or with water as a negative control. The 2 µL samples of the lysate were analyzed by the Western blot with the anti-His antibodies (Miltenyi Biotec, Bergisch gradbach, Germany). Western blots of lysates were carried out in 3 replicates and the average amounts of synthesized T7RNAPs were determined.

### 4.5. Obtaining Unnatural Templates

To obtain templates comprising the unnatural nucleotide dNaM in the second position of the V29 codon, a reverse primer containing the required substitution was synthesized (Gene Link, Orlando, FL, USA) (Table 3). In the first stage, PCR was performed with the forward primer complementary to the SP6 promoter region and the reverse primer containing dNaM in the presence of d5SICSTP (MyChem, San Diego, CA, USA). During the amplification of the template, d5SICSTP was added to the mixture of nucleotide triphosphates to a final concentration of 0.4 mM. To adjust the concentration of MgCl_2_, it was increased in the reaction mixture from 2 mM to 2.4 mM. Amplification was performed in a 50 μL mixture using Q5 DNA polymerase (NEB, Ipswich, MA, USA) and 25 pmol of each primer (glob-Nluc forward primer and Nam-Seq1 reverse primer). The amplification program consisted of 1 cycle of initial denaturation at 98 °C for 30 s of the plasmid with the Nluc gene; 25 amplification cycles—primer annealing at 60 °C for 10 s, elongation at 72 °C for 80 s and denaturation at 98 °C for 10 s. At the final stage, one cycle of DNA elongation was performed at 72 °C for 5 min. Thereafter, the 200 bp PCR product was purified in 1% agarose gel using a Qiagen kit (Qiagen, Hilden, Germany). This PCR product was added to the second PCR to produce a full-length PCR product with a reverse primer Nluc-A50 complementary to the 3′ end of the noncoding region comprising the T50 sequence. The volume of the mixture was 50 μL and it contained 25 μL (½ V) of previously purified PCR product. Amplification program: 1 cycle—98 °C, 30 sec, initial denaturation; 28 cycles—98 °C, denaturation for 10 sec. Annealing of the primer 69 °C, 10 s. Elongation at 72 °C for 160 s. Final elongation—5 min at 72 °C. Since the NaM-5SICS pair is specific [39,40], it was not eliminated during the amplification process due to DNA polymerase errors.

**Figure 4:**
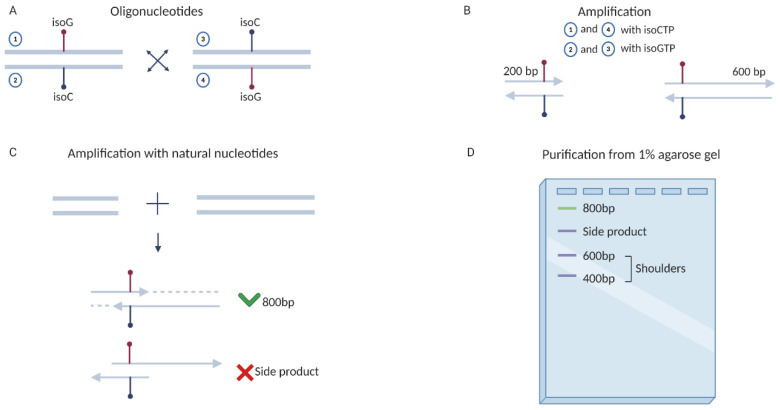
Scheme of the approach used to produce templates containing unnatural nucleotides isodG and 5-Me-isodC. (**A**) Pairs of primers complementary to each other, so that the forward primer carried 5-Me-isodC in the second position of the V29 codon, and the reverse primer carried isodG, and vice versa. (**B**) In the first round of PCR, two fragments were amplified using primers flanking both ends of the template and mutant primers carrying unnatural nucleotides. (**C**) In the second round of amplification, these PCR products were mixed without adding primers flanking ends of the template and performed 10 amplification cycles without using unnatural nucleoside triphosphates. (**D**) Single-stranded DNA molecules and unreacted fragments remaining in the reaction were removed by agarose gel electrophoresis.

Pair of unnatural nucleotides 5-Me-isodC–isodG does not reveal enough specificity which leads to the accumulation of errors with every round of amplification of approximately 2% [26,29,59]. This indicates that after each round, the unnatural pair will be partially eliminated, and after 20 cycles, a significant loss of UBP will occur, which will hinder unambiguous interpretation of the results. To overcome this limitation, we synthesized pairs of primers complementary to each other, so that the forward primer carried 5-Me-isodC in the second position of the V29 codon, and the reverse primer carried isodG, and vice versa (Figure 4). In the first round of PCR, two fragments were amplified using primers flanking both ends of the template and mutant primers carrying unnatural nucleotides (Figure 4). PCR was performed in the presence of complementary nucleotide triphosphates—in the reaction with oligonucleotides containing 5-Me-isodC, isodGTP (Jena Bioscience, Jena, Germany) was added, and vice versa, 5-Me-isodCTP (MyChem, San Diego, CA, USA) was added with a primer containing isodG. As the amplified PCR product contained a modified primer, the total loss of the template with unnatural bases was ≤2%, that is, 98% of the templates carry an unnatural nucleotide.

Amplification of fragments was performed in 50 μL of the mixture using Q5 DNA polymerase (NEB, Ipswich, MA, USA) and 25 pmol of each primer. First pair: glob-Nluc forward primer and isoC reverse primer; second pair: isoG forward primer, Nluc-A50 reverse primer. In such a case, the working chain for the T7 RNAP contains isoC. For a template with isoG decoding, the same steps were carried out, but the first pair contained the isoG reverse primer, and the second pair contained the isoC forward primer. The amplification program consisted of 1 cycle of initial denaturation of the plasmid with the Nluc gene at 98 °C for 30 s; 25 amplification cycles—primer annealing at 60 °C for 10 s, elongation at 72 °C for 80 sec and denaturation at 98 °C for 10 s. At the final stage, 1 cycle of DNA elongation was performed at 72 °C for 5 min. Then, PCR products of 200 bp and 600 bp were purified in 1% agarose gel using a Qiagen kit (Qiagen, Hilden, Germany).

In the second round of amplification, we mixed these two PCR products without adding flanking primers and performed 10 amplification cycles without using unnatural nucleoside triphosphates (Figure 4). The volume of the mixture was 100 μL. Amplification program: 1 cycle—98 °C, 30 s, initial denaturation; then 10 cycles—98 °C, 10 sec—denaturation. Annealing of the primer—69 °C, 10 s. Elongation at 72 °C for 90 s. The final extension of the DNA ends was carried out for 5 min at 72 °C. As a result, we obtained a full-length PCR product encoding Nluc containing a 5-Me-isodC–isodG pair. Since we did not use primers flanking ends of the amplified sequence, the percentage of unnatural nucleotides in the template did not change and remained at 98%. Single-stranded DNA molecules and unreacted fragments remaining in the reaction were removed by agarose gel electrophoresis (Figure 4). The resulting templates with substitutions for dNaM, 5-Me-isodC, and isodG in the lower chain were used for functional analysis of mutant T7 RNAPs in the coupled^2^ cell-free system.

## Figures and Tables

**Table 1:** Mutants of T7 RNAP, tested in the study.

Mutant	Transcription Activity	References
P266L	Increased thermal stability and promoter clearance	[53]
N433Q	Increased thermal stability	[54,55]
G542V	Involved in interactions with the 2′-hydroxyl moiety of ribonucleotides	[50]
R627S	Increased thermal stability, interactions with phosphate groups of NTPs, incorporation of ddNTP	[54,55]
S633P	Increased thermal stability	[54,55,56]
H772A		[56]
F849I	Increased thermal stability	[54,55,56]
F849A	Increased thermal stability	[54,55,56]
F849Y	Increased thermal stability	[54,55,56]
**FAL**: P266L/Y639F/H784A	Increased thermal stability, can accept 2′-O-methyl triphosphates	[31,54,57]

**Table 2:** Components of the coupled^2^ system.

Components	Final Concentration
TNT^®^ Wheat Germ Extract (Promega, Madison, WI, USA)	50%
TNT buffer (Promega, Madison, WI, USA) 25×	1×
Amino Acid mix (Promega, Madison, WI, USA) 1 mM	0.02 mM
RiboLock RNase Inhibitor (Thermo Fisher Scientific, Waltham, MA, USA) 40 U/µL	0.8 U/µL
Nluc PCR product 10 ng/µL	1 ng/µL
SP6 RNA Polymerase (Thermo Fisher Scientific, Waltham, MA, USA) 200 U/µL	16 U/µL
T7 Polymerase PCR product	10 ng/µL
Nano-Glo^®^ (Promega, Madison, WI, USA)	1%

**Table 3:** Sequences of primers.

Primer	Sequence
Forward primer (T7 RNAP)	5′-ACG CCA AGC TAT TTA GGT GAC ACT ATA GAA T-3′
Reverse primer (T7 RNAP)	5′-TTT TTT TTT TTT TTT TTT TTT TTT TTT TTT TTT TTT TTT TTT TTT TTT TTG GGC GAA TTG GCC AAG TCG GC-3′
Forward primer (Nluc)	5′-CCA GTG CCA AGC TTA ATA CGA CTC ACT ATA G-3′
Reverse primer (NLuc)	5′-TTT TTT TTT TTT TTT TTT TTT TTT TTT TTT TTT TTT TTT TTT TTT TTT TTA AAC AGC TAT GAC CAT GAT T-3′
T7_HindIII_F	5′-TTT AAG CTT GCT TTT GAC ACA ACT GTG TTT ACT TGC AAT CCC CCA AAA CAG ACA CCA TGG GAT CTC ATC ATC ATC ATC ATC ACT CTG CTG GTG AAA ACC TTT ACT TCC AGG GTG TGG GAT CCA ACA CGA TTA ACA TCG CTA AGA A-3′
T7_XhoI_R	5′-AAA ACT CGA GTT ACG CGA ACG CGA AGT CC-3′
Forward primer (T7 RNAP)	5′-ACG CCA AGC TAT TTA GGT GAC ACT ATA GAA T-3′
Reverse primer (T7 RNAP)	5′-TTT TTT TTT TTT TTT TTT TTT TTT TTT TTT TTT TTT TTT TTT TTT TTT TTG GGC GAA TTG GCC AAG TCG GC-3′
Forward primer (Nluc)	5′-CCA GTG CCA AGC TTA ATA CGA CTC ACT ATA G-3′
Reverse primer (NLuc)	5′-TTT TTT TTT TTT TTT TTT TTT TTT TTT TTT TTT TTT TTT TTT TTT TTT TTA AAC AGC TAT GAC CAT GAT T-3′
Reverse primer (V29-NaM2)	5′-TG AAA CAA ACT GGA C-NaM-C ACC TCC CTG TTC AA-3′
Forward primer (V29-iC2)	5′-TT GAA CAG GGA GGT G-iC-G TCC AGT TTG TTT CAG AAT CTC-3′
Reverse primer (V29-iG2)	5′-GAG ATT CTG AAA CAA ACT GGA C-iG-C ACC TCC CTG TTC AA-3′
Forward primer (V29-iG2)	5′-TT GAA CAG GGA GGT G-iG-G TCC AGT TTG TTT CAG AAT CTC-3′
Reverse primer (V29-iC2)	5′-GAG ATT CTG AAA CAA ACT GGA C-iC-C ACC TCC CTG TTC AA-3′

## Data Availability

The data presented in this study are available on request from the corresponding author.

## Abbreviations

CDS	CoDing Sequence
dNTPs	deoxyribonucleotide triphosphate
isoC	isocytosine (2-amino-4-ketopyrimidine)
isoG	isoguanine (6-amino-2-ketopurine)
Nluc	nanoluciferase
NTPs	ribonucleotide triphosphate
PCR	Polymerase Chain Reaction
RNAP	RNA polymerase
UBP	unnatural base pair
5′UTR	5′-untranslated regions
WGE	wheat germ extract
Wt	wild type
